# New Strategy for COVID-19: An Evolutionary Role for RGD Motif in SARS-CoV-2 and Potential Inhibitors for Virus Infection

**DOI:** 10.3389/fphar.2020.00912

**Published:** 2020-06-12

**Authors:** Shijia Yan, Haixia Sun, Xianzhang Bu, Guohui Wan

**Affiliations:** National-Local Joint Engineering Laboratory of Druggability and New Drug Evaluation, National Engineering Research Center for New Drug and Druggability (cultivation), Guangdong Provincial Key Laboratory of New Drug Design and Evaluation, School of Pharmaceutical Sciences, Sun Yat-Sen University, Guangzhou, China

**Keywords:** SARS-CoV-2, pandemic, COVID-19, drug screening, RGD, integrin

In December 2019, a pneumonia outbreak termed COVID-19 by WHO occurred in Wuhan, Hubei province, China (Chan et al., 2020). By 30 May 2020, the pandemic had caused over 6,000,000 global laboratory-confirmed infections and 364,459 fatal cases. COVID-19 is caused by SARS-CoV-2, a single-stranded positive sense RNA virus of betacoronavirus, and can result in severe respiratory diseases, such as acute respiratory distress syndrome (ARDS) (Zhu et al., 2020). The enormous economic and social impact of the SARS-CoV-2 infection makes it paramount to develop viable vaccines and antiviral drugs.

SARS-CoV-2 is an enveloped unsegmented single-stranded positive sense RNA virus, derived from the Coronaviridae family Nidovirales. We performed multiple sequence alignments of S proteins from SARS-CoV-2 and other human coronavirus, including SARS, OC43, MERS, NL63, 229E, and HKU1, using RaTG13, a Bat-SARS-like coronavirus, as a control and found that S protein of SARS-CoV-2 maintains the highest homology (96%) sequence with RaTG13, and 75% of homology sequence with SARS-CoV, but is significantly different from other human coronavirus (**Figures 1A, B**). The full-length genome sequences of SARS-CoV-2 share a 79.5% sequence identity to SARS coronavirus (SARS-CoV). It was reported that, similar to SARS-CoV, the S glycoprotein of SARS-CoV-2 binds the cellular receptor angiotensin-converting enzyme 2 (ACE2) in hosts to mediate fusion of the viral and cellular membranes (Lu et al., 2020; Wrapp et al., 2020)). The binding affinity between S_SARS-CoV-2_ protein and ACE was suggested to be 10 to 60 nM, 10 to 20-fold higher than S_SARS-CoV_ (Lan et al., 2020; Yan R. et al., 2020), but the underlying mechanism remains inconclusive. Variations of crucial residues in the S_SARS-CoV-2_ protein and their counterpart receptors may contribute to the high transmission efficiency. SARS-CoV-2 produced an evolutionary mutation of K403R compared with S_SARS-CoV-2_ protein, forming an adjacent RGD motif (Arg-Gly-AsP) at the interaction surface (Luan et al., 2020; Sigrist et al., 2020; Tresoldi et al., 2020; Yan S. et al., 2020). Point mutation of the second codon of “ACA” into “AGA” at site 403 creates an Arg from Thr compared with the sequence in RaTG13, while the counterpart in SARS is “AAG” coding for Lys (**Figures 1C, D**).

Integrin is a transmembrane heterodimeric protein comprising of α and β subunits, many of which recognize the RGD motif displayed on the exposed loops of viral capsid proteins. The RGD motif is the cell attachment site, which can recognize integrin of various epithelial cells to promote cell adhesion and virus internalization by activating transducing pathways involving phosphatidylinositol-3 kinase (PI-3K) or mitogen-activated protein kinase (MAPK) (Ruoslahti, 1996). Meanwhile, ACE2 was previously found to bind with integrin, regulates the cardiac remodeling signaling pathway, and affects cell survival and proliferation (Lin et al., 2004; Clarke et al., 2012). Integrin β1 can regulate CCL2 levels in alveolar epithelial cells, recruiting monocytes to induce an inflammatory response (Plosa et al., 2020), suggesting that the RGD sequence of the S_SARS-CoV-2_ protein may be recognized by integrin in alveolar epithelial cells to accelerate the infection process.

It has been confirmed that the S_SARS-CoV-2_ protein adopts a similar conformation and interaction mode to that of S_SARS-CoV_ when interacting with ACE2 (Xu et al., 2020). The spatial structure of the RGD motif (403-405) is located outside of the S protein and adjacent to its interaction interface with ACE2 (**Figure 1E**), defining a small loop between a β-strand and an α-helix. Previous studies reported that the S protein processes a dynamic prefusion conformation during fusion into the host cell membrane (Li, 2016; Wrapp et al., 2020). When the receptor binding domain (RBD) of S1 subunit undergoes hinge-like conformational shifts, the change exposes or hides the key region of binding domain to access ACE2 by controlling the “up” and “down” conformation, exposing the RGD motif to the surface of the host cell membrane in conjunction with the key binding region. Once interacting with integrin, ACE2 may be recruited to the binding complex, facilitating the invasion of the virus. Another possible mechanism is proposed that the RGD motif may bind to integrins parallelly or sequentially in an ACE2-independent manner, which is supported by the role of ACE2 serving as a cell adhesion substrate and regulating integrin signaling (Clarke et al., 2012). However, Luan et al. held a contrary view that integrin can inhibit receptor targeting of S proteins from SARS-CoV-2 by shielding both S protein and ACE2, since there would be no space for ACE2 to contact with S if associated with integrin. In general, it is hypothesized that the RGD motif may play an important role in promoting rapid transmission in SARS-CoV-2.

Though several articles have reported the RGD motif and its potential role, no drugs have been investigated in preclinical studies or clinical trials (Sigrist et al., 2020; Tresoldi et al., 2020). Based on this rationale, high-throughput virtual screening searching for potential therapeutic drugs targeting interaction of S_SARS-CoV-2_ protein with both ACE2 and integrin were performed according to the hypothesis. Our compound libraries include FDA-approved drug entities (2040 species), our own medicine food homology natural products derivatives entities (1500 species), and cyclic peptides entities (230 species), along with virtual bioactive and natural products libraries (**Figure 1F**). The S_SARS-CoV-2_ structure extracted from the recent crystal structure of S protein/human ACE2 complex (NMDCS0000001) were adopted, choosing the key residues in the S protein interface (Q493, Y495, Q498, N501, and Y505) and R403, D405 as the potential binding site (**Figure 1G**) for virtual screening by Sybyl X using the Surflex-Dock Geom (SFXC) approach.

**Figure 1:**
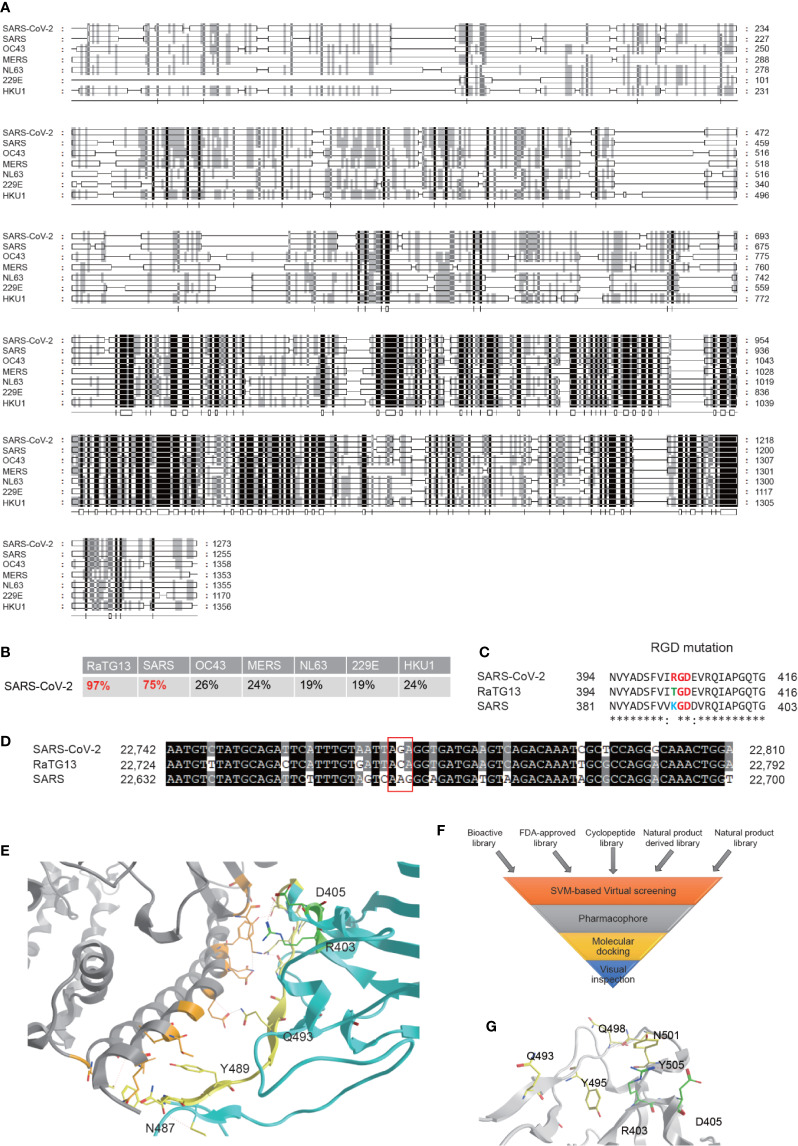
Bioinformatics analysis of the S protein of the SARS-CoV-2 and identification of potential inhibitors for its interaction with ACE2. **(A)** Amino acid sequence alignment of the S proteins in the coronaviruses infecting humans, including SARS-CoV-2, SARS, OC43, MERS, NL63, 229E, and HKU1 strains. **(B)** Homology of SARS-CoV-2 to other coronaviruses based on the S protein alignment. RaTG13, a Bat-SARS-like coronavirus, is used as a control. **(C)** Evolution of the RGD motif in the S protein of SARS-CoV-2 compared with the S protein of SARS-CoV. **(D)** Nucleotide mutation produces RGD in SARS-CoV-2. **(E)** ACE2 binding interface regions of the S protein of SARS-CoV2. Location of the RGD motif was shown in green, and the yellow residues are the interaction surfaces binding with ACE2. **(F)** Flowchart of interaction surface structure-based virtual high-throughput screening. **(G)** The key residues in the S protein interface and the RGD motif were chosen as the potential binding site to generate the protomol for virtual screening by using Surflex-Dock Geom (SFXC) approach.

The representative agents hit included: Nadide, Losartan, and Adenosine phosphate from the FDA-approved drug library; Difludionone-119 and Methyl-benzyloxychadone-844 from the natural products derivatives library; GR8-1 [Cyclo (f-P-V-R-f-P-R-L-)], MGR-7 [Cyclo (L-f-P-V-R-L-f-P0-V-R-)], and GR6-2 [(Cyclo (R-f-P-R-f-P-)) from our own cyclic peptide library; and S-9′″-Methyllithospermate B and the S-Leonurine from the bioactive and natural product library respectively(Yan S. et al., 2020). These compounds were well docked into the pocket formed by the selected key residues and RGD motif by Hydrogen bonds, and/or π-π/p-π interactions respectively, providing potential antiviral drug candidates for COVID-19.

Particularly, Nadide was scored with a high grade (10.7719), which is superior to other hits, implying it may serve as a promising drug candidate for COVID-19. Nadide is a dinucleotide of adenine and nicotinamide and has coenzyme activity in redox reactions, acting as a donor of ADP-ribose moieties (Bertoldo et al., 2020). It was postulated that supplement of nicotinamide may resist viral infection through innate immunity (Heer et al., 2020). SARS-CoV-2 infection can strikingly dysregulate the nicotinamide adenine dinucleotide (NAD) gene set by inducing a set of poly ADP-ribose polymerase (PARP) family enzymes required for the innate immune response. Overexpression of PARP10 induces a significant decrease in host cell NAD while boosting NAD through the nicotinamide and nicotinamide riboside kinase pathways, can restore antiviral PARP functions to support innate immunity to SARS-CoV-2, which provides a clue that Nadide may play a role in preventing COVID-19. Further *in vivo* study is needed to validate the effects of Nadide to block the interaction between the RGD motif and ACE2 protein.

Losartan is another promising potential drug candidate for COVID-19. ACE2 is a carboxypeptidase, negatively regulating Ang II production and counterbalancing the function of ACE. Losartan, a selective and competitive nonpeptide Ang II receptor antagonist, was known to block the vasoconstrictor and aldosterone-secreting effects of Ang II and interact reversibly with AT1 and AT2 receptors. It was postulated that SARS-CoV may promote severe acute lung injury pathogenesis through increased AngII production and functional alterations of the renin-angiotensin system, and the lung failure can be rescued by inhibition of AT1R (Kuba et al., 2005). Therefore, it is reasonable to presume that SARS-CoV-2 Spike may also exaggerate acute lung failure through the allied mechanism of SARS-CoV which deregulates the renin-angiotensin system, and can be rescued by inhibition of AT1R. Since losartan is a commonly used antihypertensive drug in clinical practice, its toxicological and pharmacokinetic properties have been fully studied and confirmed by a large amount of clinical data. If proven effective against the SARS-CoV-2 infection, it could be reassessed as an antiviral drug and significantly shorten the research cycle for drugs.

Since coronaviruses are under extensive mutagenesis and the mutation in key proteins are crucial to the virus, the potential clinical significance of the S protein harboring the RGD motif in SARS-CoV-2 is notable. Compared with SARS-CoV, SARS-CoV-2 has comparable, even higher, transmissibility that urges us to uncover its infection mechanism and to develop specific drugs against SARS-CoV-2 to alleviate the current pandemic (Chen et al., 2020; Li et al., 2020). It was hypothesized that the RGD motif on the S glycoprotein may bind to the integrin on the surface of host cells, resulting in higher affinity with the host cells in comparison with SARS-CoV. Further investigations are needed to verify and determine the specific subtype of integrins to interact with the S_SARS-CoV-2_ protein (Stewart and Nemerow, 2007). Meanwhile, infection blockers can be designed to be highly compatible with S protein to block either ACE2 binding or integrin binding. Griffithsin, for example, has been previously reported to bind to oligosaccharides of various viral glycoproteins, which can be reassessed as a treatment (Zumla et al., 2016; Lee, 2019). Multiple RGD-related peptides are currently being used in clinical trials; for example, 18F-αvβ6-BP is currently used to detect lung damage where αvβ6 is a RGD-recognizing integrin (NCT04376593). In our screening we also show several potential cyclic peptides as transmission blockers for SARS-CoV-2, but their effects are currently under investigation (Yan S. et al., 2020). Collectively, in our screen, Nadide was shown to block the interaction of the RGD motif and its unknown integrin counterpart simultaneously, serving as a promising potential drug candidate for COVID-19. Integrin-targeted drugs might modulate virus-ligand affinity and signaling of SARS-CoV-2, and provide a new strategy in controlling COVID-19.

## Author Contributions

GW and XB initiated the concept and design of the study. XB, GW, SY, and HS performed the analysis. GW and SY wrote the draft. GW and XB reviewed and revised the manuscript. GW and XB supervised the study.

## Conflict of Interest

The authors declare that the research was conducted in the absence of any commercial or financial relationships that could be construed as a potential conflict of interest.
